# Cardiovascular Manifestations of Pseudoexfoliation Syndrome: A Narrative Review

**DOI:** 10.7759/cureus.51492

**Published:** 2024-01-01

**Authors:** Rajal R Bora, Roshan Prasad, Swapneel Mathurkar, Kashish Bhojwani, Akshansh Prasad

**Affiliations:** 1 Medicine, Jawaharlal Nehru Medical College, Datta Meghe Institute of Higher Education and Research, Wardha, IND; 2 Ophthalmology, Jawaharlal Nehru Medical College, Datta Meghe Institute of Higher Education and Research, Wardha, IND; 3 Pathology, Jawaharlal Nehru Medical College, Datta Meghe Institute of Higher Education and Research, Wardha, IND; 4 Medicine, Banaras Hindu University, Varanasi, IND

**Keywords:** loxl-1 gene, cardiovascular disease, cerebrovascular disease, homocysteine, manifestations, pseudoexfoliative material, glaucoma

## Abstract

Pseudoexfoliation syndrome (PEX) is a long-term, age-related extracellular matrix condition that causes aberrant fibrillary pseudoexfoliative material (PXM) to accumulate in various body tissues. The anterior portion of the eye is where this disorder most frequently presents. It affects the entire body. Most frequently, it is seen in older people, usually those over 50. Fibrillar deposits are a symptom of the pseudoexfoliation syndrome and are found in the anterior part of the eye. Deposition of fibrillary white flaky material is seen. The lens capsule, cornea, ciliary epithelium, lens epithelium, iris pigment epithelium, zonules, orbital soft tissues, trabecular meshwork, iris blood vessels, and iris stroma have all been reported to show such depositions. The skin, heart, lungs, liver, kidneys, and other organs have also been reported to contain these deposits.

Asymmetrical and bilateral illnesses are both possible. Myocardial infarction, cerebrovascular accidents, and systemic hypertension have all been linked to it. The pseudoexfoliative condition was first reported with the characteristic findings of white or grey flakes on the anterior lens capsule, the prevalence of glaucoma rising with age, and its presence in about 50% of eyes. A few decades later, the term pseudoexfoliation was given to differentiate it from the true exfoliation syndrome. True exfoliation syndrome is characterized by lamellar delamination of the lens capsule and is caused by exposure to infrared radiation. It is commonly seen in glassblowers.

Age is a risk factor for PEX once a person reaches 70. Symptoms of PEX include elevated intraocular pressure, peripapillary transillumination deficiencies, potential glaucomatous optic nerve damage, poor dilatation, Sampaolesi line, and fibrillar white flaky deposits along the pupillary border. Meanwhile, fibrillar white flaky deposits on the anterior lens capsule (Hoarfrost Ring) and pigment dispersion syndrome are not pathognomonic.

## Introduction and background

Pseudoexfoliation syndrome (PEX) is diagnosed due to the increasing buildup of an aberrant extracellular fibrillar substance, pseudoexfoliative material (PXM) in tissues outside the eye, such as connective tissues and skin in the regions of numerous abdominal organs and anterior components of the eye. PEX is typically observed in elderly people. The primary fibrillar component of PXM is immersed in sluggish ground material [1].

The anterior chamber and its angle, the centre disc, the fibres of ciliary zonules, the iris, the pupil, the trabeculae, and the cornea, along with ciliary processes, can all be observed to have a white, dandruff-like substance. On all surfaces, PXM is seen histopathologically as bush-like eosinophilic excrescences. Age has a significant impact on PEX prevalence [2]. PEX evolves with ageing and often progresses to exfoliation glaucoma in the elderly. This is caused by the accumulation of extracellular material and other debris and the age-related shortening of the outflow channels to Schlemm’s canal’s inner wall. In younger people, this collection can effortlessly be removed through the trabecular meshwork, but in elderly people, it gets lodged in the trabecular meshwork. The PXM degrades tissues and further obstructs aqueous humour outflow routes when lodged in significant amounts close to the endothelial cells of the trabecular meshwork and Schlemm’s canal. The quantity of entrapped deposits has been directly connected with rising intraocular pressure, which leads to open-angle glaucoma (OAG) [3].

Additionally, it is linked to the development of cataracts as well as intraoperative issues such as zonular or posterior capsule rupture, a poor ability to dilate the pupil, vitreous loss, fibrinoid response, subluxation of the intraocular lens that is implanted, and decompensation of the corneal endothelium. The same deposits are also seen in other abdominal organs, like the meninges, brain, heart, lungs, arteries, kidneys, and gallbladder, though their practical importance is unknown. These deposits have also been found in the part of the eye’s frontal area [4].

A study showed that the occurrence of bilateral PEX is approximately 1.6x greater in comparison to unilateral PEX, and it rose with increasing age from 52 per cent in the seventh decade to 77 per cent in patients over the age of 80. In actuality, fibres of the PEX eye have been visualised on an electron microscope in the conjunctiva and other parts of the orbit in the control group’s eyes, indicating that the pathology is relatively present in both eyes with an unequal appearance. Additionally, in the iris of every other eye that is unaffected, immunohistochemical and ultrastructural results that are particular to PEX fibres have been found [5].

In a cross-sectional study by Andrikopoulos et al. [6] conducted in Greece in 2003, 50 (53.8%) of the 371 participants in the study had bilateral PEX, and 93 (25.1%) of them had glaucoma. Amidst the 225 people with unilateral PEX, 17.3% of patients (39) were also found to be suffering from OAG, 23.1% (9) of them had bilateral glaucoma, and only two of them presented with glaucoma in the eye that wasn’t affected by PEX. These findings raise the possibility of a connection between the quantity of greyish-white deposits and their role in initiating OAG. Of the total 596 patients with PEX, 304 (51%) were female and 292 (49%) were males [6]. In another prevalence study by Arnarsson, conducted in Iceland in 2009, PEX prevalence was found to be 1% more in females than in males [7]. Studies reporting on the association of PEX with gender have found nearly the same incidence and prevalence in both genders. Thus, the link between PEX and gender distribution remains unclear.

## Review

Methodology

We conducted a thorough literature search to find pertinent papers for our review article. We searched the database of PubMed and Google Scholar using the keywords "glaucoma", "pseudoexfoliative material", "manifestations", "cerebrovascular disease", "homocysteine", "cardiovascular disease", and "LOXL-1 gene". We included only the articles which were written in English and have ensured the inclusion of recently published articles, which provide value for comprehensive research. Manual searches of reference lists were also carried out to find other pertinent studies. Our inclusion criteria incorporated studies focusing on the cardiovascular manifestation of the PEX, its pathogenesis, cerebrovascular manifestations, homocysteine, and the lysyl oxidase-like 1 (LOXL-1) gene. Original research papers as well as review papers, were taken into consideration for inclusion. Articles written in languages other than English and those with missing or inaccessible full texts were excluded. We ensured that only those research papers that fit the preset criteria were included. Titles, abstracts, and full-text papers were screened during selection. The authors reached a consensus to settle disagreements on the choice of studies.

Pathogenesis

The building of PEX deposits in arteries and veins, leading to increased resistance by vessels and impaired vascular flow are only a few of the pathogenic pathways proposed to explain why people with PEX have a higher risk of developing vascular illness. Deposits of PEX have been proposed as a mechanism for atherosclerosis and the development of thrombi, and thus, result in coronary events by insulting the endothelium of vessels and functions of smooth muscle. Apolipoprotein A, homocysteine, and other cardiovascular risk indicators have moreover been detected in increased levels in persons suffering from PEX in contrast to normal subjects without PEX in the past, primarily as components of relatively small clinical trials [8].

Additionally, it has been proposed that factors not involving genetic involvement, such as exposure to UV light, nutritional factors, biological agents, injury, oxidative stress, inflammation, and hypoxia, function as co-modulating external factors. It appears that a local shift in the equilibrium amongst matrix metalloproteinases and tissue inhibitors of metalloproteinases is what is causing the malfunction of the fibrotic matrix along with the deposition of PXM. Transforming growth factor 1 (TGF-1), growth factors (GFs), profibrotic cytokines (Interleukin-6), as well as improper protection of cellular systems with elevated cellular and oxidative stress, are thought to be involved [9].

Additionally, low-grade chronic inflammatory processes, cross-linking mechanisms, aggregation of stressed, misfolded proteins and ischemia/hypoxia have all been suggested as contributing factors. PEX material appears to be a complex of extremely interconnected glycoprotein proteoglycan made up largely of stretchable micro-fibrillar proteins like fibrillin-1 and latent transforming growth factor binding protein, chaperons like clusterin, as well as enzymes which promote cross-linking like LOXL-1. With a late onset and incomplete penetrance, PEX is presented as an autosomal dominant characteristic. The discovery of the LOXL-1 gene, found to be present over chromosome 15q24 in the expatriate Icelandic community, having the phenotype of PEX, was a significant advance in our understanding of the disease. Several research in various groups verified the genetic propensity of carriers of LOXL-1 to PEX. Apart from the LOXL-1 gene, clusterin and lysosomal transport regulators are also responsible [10].

Assisting in the production of elastin fibres in the matrix outside the cell, LOXL-1 attaches to tropoelastin monomers. The current information states that low expression of LOXL-1 in the latter phases of this pathology affects the metabolism of elastin and stimulates elastotic processes, which dispose them for the onset of OAG and added diseases. The protein's localised distribution and surface association with fibres suggest a crucial involvement in developing aggregated filamentous proteins. Given that LOXL-1 acts as an amino oxidase, it may stick to the elastic fibres that will eventually become a component of the PEX material once elastin is cross-linked. Data indicates that the upregulation of LOXL-1 in the initial phase of fibrogenesis of PEX causes the accumulation of atypical deposits [11,12].

PXM

PXM, according to studies utilising transmission electron microscopy, is a fibrillar substance. A PXM fibril comprises individual microfibrils that are 8 to 10 nm in diameter and are generated through lateral aggregation. Furthermore, the PXM fibrils usually conceal their microfibrillar nature due to their electron-dense amorphous coating. In PXM fibrils, immunohistochemical studies have discovered the presence of latent transforming growth factor binding proteins 1 and 2 (LTBP-1 and LTBP-2) as well as tropoelastin, microfibril-associated glycoprotein, fibronectin, elastin, emilin, heparan sulphate proteoglycan, amyloid P, fibrillin-1, and vitronectin. The existence of fibrillin-1, fibrillin-2, vitronectin, and amyloid P-component has been confirmed by tandem mass spectrometry and liquid chromatography. Additionally, it made it possible to pinpoint the components of PXM microfibrils, including desmosomal cadherins (desmo-colin-2), tissue inhibitors of metalloproteinases (TIMP3), ADAMTS-8, 18, and 19 of the ‘a disintegrin and metalloproteinase’ (ADAM) family, serum amyloid protein, laminin, proteoglycans syndecan-3 and fibronectin [13].

**Figure 1:**
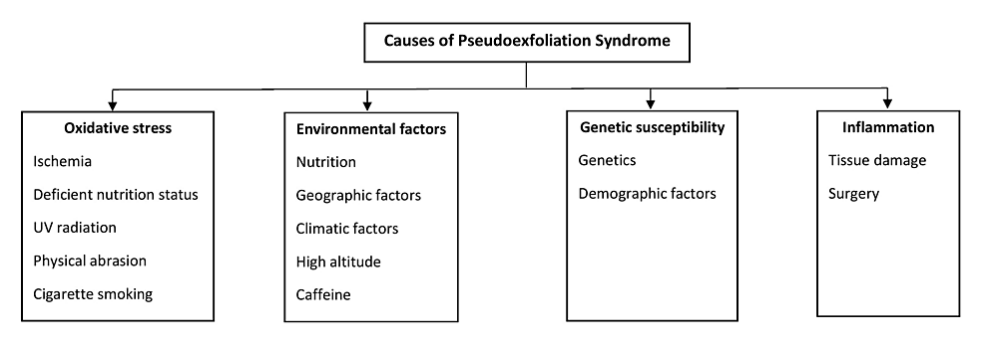
Causes of pseudoexfoliation syndrome Image Credit: Author Roshan Prasad using information from [14].

It is uncertain whether excessive synthesis or insufficient breakdown is to blame for PXM accumulation. It was found that transglutaminase 2 and other PXM components like LTBP-1 and 2, as well as elastic microfibril components like fibrillin-1, were found in tissues in the anterior segment of the eye, where PXM is commonly observed to be elevated at both the messenger RNA (mRNA) and protein levels. This implies that the primary cause is excessive de novo synthesis. However, the probability of incorrect PXM degradation is supported by a documented imbalance between matrix metalloproteinases (MMPs) and their TIMP3. Likely, excessive synthesis and insufficient degradation work together to cause PXM buildup [14]. Apart from genetics, Figure 1 shows the other causes of PEX.

Clusterin

Clusterin is a glycoprotein consisting of two chains named alpha clusterin and beta clusterin, which are present in body fluids. The function of clusterin is lipid transport and is usually released during cellular stress; it has a protective function of reducing oxidation by binding to misfolded protein. Studies on clusterin have also suggested that PXM buildup could occur if there is a deficit. Clusterin mRNA is produced by most tissues and ocular cells, especially in the epithelium of the ciliary process [15]. At the same time, the protein is frequently found in extracellular structures like stromal fibres and ocular basement membranes. Clusterin has moreover been detected in the iris and optic nerve. Regardless of glaucoma, PEX eyes displayed significant downregulation of clusterin mRNA in all tissues of the anterior segment compared to non-diseased eyes and even in eyes with primary OAG (POAG). A considerable decrease in clusterin levels was also seen in the PEX eye [16].

Manifestations in the eye

In the lens, the most reliable and significant diagnostic indicator of PEX is an accumulation of greyish-white flakes on the anterior part of the lens surface. The traditional arrangement comprises three areas: a disc at the centre, which is nearly equal to the diameter of the pupil; the third and exteriormost area, which is granulated and also frequently found layered; and a clean space between the two. An early and well-known clinical characteristic of PXM in the iris is iris alternations [17]. Exfoliation debris is most noticeable next to the lens at the pupillary border. The iris blood vessels might get destroyed and are frequently constricted. The vessel wall's cells are entirely degenerate in advanced stages. In the cornea, flakes of PXM may be present. The epithelium may have a diffuse, non-specific pigmentation that occasionally resembles a Krukenberg spindle. The Schwalbe's line is where pigment is typically deposited, though it can also appear as curvy lines or the lines in front of the Schwalbe's line [18].

PEX can be diagnosed by slit lamp examination by the characteristic grey-white deposits or flaky white deposits near the papillary edges on the lens's anterior surface, separated from a girdle of deposits on the lens periphery by a clear zone. Current research with electron microscopy supports the hypothesis that PEX syndrome is a generalised bilateral illness with clinically evident asymmetry manifestation, whereas earlier, it was believed that PEX accumulation can be found on one or both sides [19].

Patients with deposits of PEX material have also been reported to experience posterior synechiae, pseudo uveitis, keratopathy, loss of normal radial iris arteries, and accelerated cataract development. Using slit lamp observations, PEX is diagnosed by looking for the characteristic grey-white flakes at the papillary margin or deposits in the core of the anterior surface of the lens capsule, separated from a peripheral griddle of deposits by a clear zone [20]. The hypothesis suggesting PEX of generalised illness present in both eyes with obvious asymmetry expression is supported by new investigations based on electron microscopy, contrary to earlier theories that deposits of PEX could be detected in one eye or both eyes [21].

PEX and the cardiovascular (CVS) system

After multivariate adjustment, which considered age, gender, presence or absence of glaucoma, and other dangerous vascular signs, PEX was substantially related to hypertension, angina, or a history that combined angina, myocardial infarction, or stroke. Despite mounting proof that PEX is a standalone risk factor for (CVS) disease, a recent study found that aortic aneurysm, coronary artery disease (CAD), and peripheral artery disease were not significantly associated with PEX [22].

**Figure 2:**
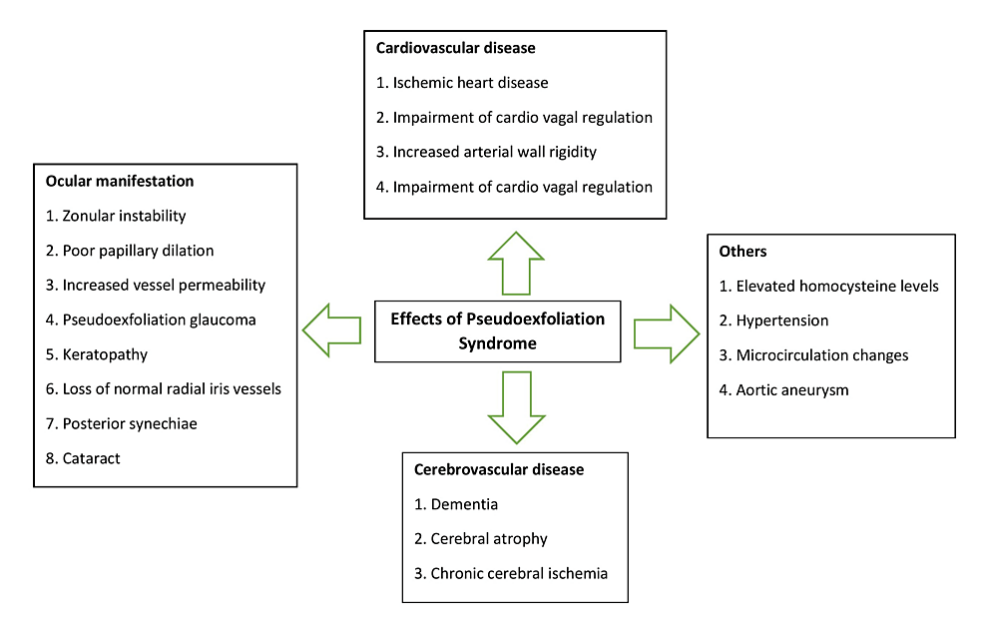
Manifestations of pseudoexfoliation syndrome Image Credit: Author Roshan Prasad with information from [21].

This was linked to the impact of vessel wall elastosis. Citirik et al. found a link between PEX and CAD. Their study on 50 patients with CAD, diagnosed by coronary angiography and 50 controls with normal coronary angiographic findings, concluded that PEX was significantly associated with CAD (P=0.001) [2]. PEX and the presence of CAD have a beneficial relationship. Arrhythmia has been observed to be more prevalent in PEX [23]. It affects the CVS system and various other body systems, as shown in Figure 2 [21].

Anomalies in the cerebrovascular flow

According to reports, patients with transient ischemia events have a significant incidence of PEX syndrome. Additionally, compared to control subjects, patients who have been diagnosed with PEX, along with the presence of glaucoma, had a significantly greater prevalence of white matter hyperintensities (ischemic alterations) as assessed by MRI. According to studies, people with PEX and PEX glaucoma had lower middle cerebral artery blood flow velocities and less blood flowing through their regional brains [24].

Additionally, individuals with PEX with glaucoma had a greater prevalence of brain illnesses than patients with POAG, including senile dementia, cerebral atrophy, and chronic cerebral ischemia. The same study found that patients with pseudoexfoliative glaucoma were likelier than those with POAG to experience acute cerebrovascular disease. PEX syndrome and Alzheimer’s disease have also been related in some research, but not all [25].

Vascular manifestations

In PEX syndrome, severe CVS disease symptoms like ischemia and reduced blood flow have frequently been observed. The pathophysiology of CVS problems in PEX people may be related to the aggregation of PEX material inside the vessels, which is responsible for the rise in vascular resistance and a decrease in blood flow, arterial dysregulation, and disturbed parasympathetic vascular modulation [26].

It has been hypothesised that increased aortic thickness in PEX patients is a factor in the cases' higher risk of CAD. Decreased distensibility with increased stiffness in the common carotid artery, along with disturbed parasympathetic vascular control linked with higher homocysteine levels in plasma in PEX or PEX associated with glaucoma than in the control group, were also reported by Visontai et al. using the ultrasound wall tracking system [27]. Other investigations that compared controls to patients with PEX syndrome found that controls had thicker carotid intima-media and lower cardiac peak systolic tissue Doppler imaging velocities. However, there was only a minor correlation between PEX and carotid plaque measures [28].

The increased risk of CAD in PEX patients has been linked to greater aortic stiffness, which may be at least largely to blame. More stiffness and less distensibility in the common carotid artery, in addition to disturbed parasympathetic vascular regulation linked to higher homocysteine levels in plasma in PEX/PEX glaucoma than the control group, was also reported by Visontai et al. utilising the ultrasound wall tracking method [27]. Other investigations that compared sufferers of PEX to controls found that patients with PEX syndrome had lower cardiac peak systolic tissue Doppler imaging velocities and thicker carotid intima-media. In contrast, there was only a minor correlation between PEX and carotid plaque readings [29].

Patients with PEX have also been reported to have impaired baroreflex sensitivity, parasympathetic cardiovascular control, and pulse wave velocity. Arterial stiffening is a sign of a higher risk of cardiovascular illness, and metabolic syndrome, hypertension, heart failure, and myocardial infarction have all been linked to diminished baroreflex sensitivity. Additionally shown were reduced cutaneous capillary blood flow, altered responses to cold and heat, and no change in plasma endothelin-1 levels. Further, elevated levels of cardiovascular risk factors such as homocysteine, apolipoprotein A, and lipoprotein (a), along with reduced brachial artery dilatation with increased carotid intima-media thickness, were discovered in PEX patients [30].

PEX may be a risk factor for peripheral vascular disease because PEX participants had significantly lower ankle brachial indices than controls. Table 1 shows the classification system of Keith, Wagener, and Barker, which was used to describe vascular alterations in the fundus [23].

**Table 1:** Classification system to describe vascular alterations in the fundus Table created by the authors using information from [23].

Group	Characteristics
I	Minimal retinal artery narrowing.
II	Retinal artery narrowing, in addition to regions of localised constriction and nicking of the aorta.
III	It includes the anomalies present in the above two groups and haemorrhages of the retina, hard exudation, and cotton-wool patches.
IV	It includes the anomalies seen in the above four groups and optic nerve head enlargement.

Although PEXS is largely an eye condition, aberrant PEX material has also been found to accumulate in tissues outside the eye, like the heart, lungs, kidneys, liver, gall bladder, and brain. The atypical PXM Is normally seen in the tissue of the aforementioned organs when extraocular involvement develops, with a preference for the blood vessel margins. This finding reveals that the defective PEX material results from an extracellular matrix disorder that may affect fibroblasts and smooth, cardiac, and striated muscle cells [31]. In addition, several reports suggested a link between PEX and conditions that affect the heart and brain, including Alzheimer’s disease, SNHL, which is sensorineural hearing loss, an abdominal aortic aneurysm, asymptomatic myocardial dysfunction, angina pectoris, and transient ischemic attacks. Acute (haemorrhages, emboli, thrombi) and persistent cerebrovascular events, including cerebral shrinkage, cerebral ischemia, and senile dementia, are frequently found among PEX patients [32].

Elevated homocysteine

A distinct risk element for CVS disease is homocysteine. It is linked to vascular damage and raises the risk of venous thrombosis, CAD, and stroke. Dysfunction of the endothelium, aggregation of platelets, and disruption of clotting factors are examples of potential modes of action. Additionally, elastolysis, oxidative stress, and changes to the extracellular matrix of various tissues (mostly arteries) may be involved [33].

Due to its ability to worsen the abnormal accumulation of matrix in PEX patients, hyperhomocysteinemia is thought to be a major contributing factor in increased vascular risk. PEX and PEX glaucoma patients have elevated plasma homocysteine levels. Homocysteine levels in patients with PEX glaucoma were found to be higher in their tears than in their aqueous humour, which was either higher or unaltered. Pyridoxine, folate, and cobalamin are involved in the metabolism of homocysteine and are detrimentally associated with whole plasma homocysteine levels. It is known that PEX glaucoma patients have lower homocysteine levels, albeit a differentiating difference between PEX and control groups was not observed in a different study [31].

Due to its propensity to create aberrant matrix accumulation in PEX patients, hyperhomocysteinemia has been proposed as a potential root of higher vascular risk. Sufferers with PEX and PEX glaucoma have been reported to have elevated plasma homocysteine levels. Homocysteine levels are high or unaltered in the aqueous humour of PEX glaucoma patients but elevated in their tears. Although they are similar between patients and control groups in a different investigation, vitamins pyridoxine, cobalamin, and folate, the ones which are critical for the metabolism of homocysteine and inversely linked with the overall homocysteine levels in the plasma, are lowered in patients of PEX glaucoma [34].

Additionally, vascular endothelial cells’ decreased LOXL-1 activity and expression have been linked to hyperhomocysteinemia. Endothelial dysfunction, which characterises earlier phases of the atherosclerotic process, has been linked to LOXL-1 downregulation. Additionally, it has been suggested that single nucleotide polymorphisms (SNPs) in the LOXL-1 gene, connected to PEX, may be associated with spontaneous cervical artery dissection. PEX impairs endothelium function and results in structural malformations of vessel walls. In the serum of PEX patients, the investigators found elevated levels of homocysteine, lipoprotein (a), and apolipoprotein A. They recommended that these sufferers be assessed for these increased CVS risk factors. The generation of the exfoliation material was associated with an increase in plasma homocysteine content and impairment to the function of the major arteries [35].

Coronary aneurysm

There has been talk of systemic macro and microcirculation impairment in PEX patients. Although several variables contribute to the development of abdominal aortic aneurysms, atherosclerosis has been linked to their occurrence. It has been suggested that PEX syndrome and abdominal aortic aneurysms are related., Histopathological analysis of samples from walls of the aorta from individuals with ocular PEX suggested tunica intima elastosis and fibrosis as well as an enormous amount of buildup of PXM in the adventitial and subendothelial layer of connective tissue. Although other investigations failed to find a significant correlation, abdominal aorta aneurysms are present more commonly in PEX patients compared to the control group [36].

## Conclusions

PEX is an accumulation of fibrous substances in the connective tissues of extraocular tissues and other organs such as the heart, lungs and other organs. It is a disease of old age. It usually starts in the anterior segment of the eye. Several factors must be considered in patients with PEX, including genetic or non-genetic causes. The genetic factor is a mutation in the LOXL-1 gene and the non-genetic cause is exposure to ultraviolet (UV rays), alcohol consumption, and dietary factors. It is diagnosed by slit lamp visualisations of white powdery deposits. The treatment of PEX is beta blockers, alpha receptor agonists, carbonic anhydrase inhibitors, and prostaglandin analogues. The complications of PEX are glaucoma, cataracts, and various systemic manifestations. It is the major cause of pseudoexfoliative glaucoma, which is a severe form of POAG. PEX is an ocular disease with systemic manifestation. The main clinical feature of PEX is the deposition of white dandruff-like material on the lens and anterior chamber.
